# bootRanges: flexible generation of null sets of genomic ranges for hypothesis testing

**DOI:** 10.1093/bioinformatics/btad190

**Published:** 2023-04-12

**Authors:** Wancen Mu, Eric S Davis, Stuart Lee, Mikhail G Dozmorov, Douglas H Phanstiel, Michael I Love

**Affiliations:** Department of Biostatistics, University of North Carolina, Chapel Hill 27514, United States; Curriculum in Bioinformatics and Computational Biology, University of North Carolina, Chapel Hill 27514, United States; Genentech, South San Francisco, Western California 94080, United States; Department of Biostatistics, Virginia Commonwealth University, Richmond, VA 23284, United States; Department of Pathology, Virginia Commonwealth University, Richmond, VA 23284, United States; Curriculum in Bioinformatics and Computational Biology, University of North Carolina, Chapel Hill 27514, United States; Thurston Arthritis Research Center, University of North Carolina, Chapel Hill 27514, United States; Department of Cell Biology and Physiology, University of North Carolina, Chapel Hill 27514, United States; Lineberger Comprehensive Cancer Center, University of North Carolina, Chapel Hill 27514, United States; Curriculum in Genetics and Molecular Biology, University of North Carolina, Chapel Hill 27514, United States; Department of Biostatistics, University of North Carolina, Chapel Hill 27514, United States; Curriculum in Bioinformatics and Computational Biology, University of North Carolina, Chapel Hill 27514, United States; Lineberger Comprehensive Cancer Center, University of North Carolina, Chapel Hill 27514, United States; Department of Genetics, University of North Carolina, Chapel Hill 27514, United States

## Abstract

**Motivation:**

Enrichment analysis is a widely utilized technique in genomic analysis that aims to determine if there is a statistically significant association between two sets of genomic features. To conduct this type of hypothesis testing, an appropriate null model is typically required. However, the null distribution that is commonly used can be overly simplistic and may result in inaccurate conclusions.

**Results:**

*bootRanges* provides fast functions for generation of block bootstrapped genomic ranges representing the null hypothesis in enrichment analysis. As part of a modular workflow, *bootRanges* offers greater flexibility for computing various test statistics leveraging other Bioconductor packages. We show that shuffling or permutation schemes may result in overly narrow test statistic null distributions and over-estimation of statistical significance, while creating new range sets with a block bootstrap preserves local genomic correlation structure and generates more reliable null distributions. It can also be used in more complex analyses, such as accessing correlations between cis-regulatory elements (CREs) and genes across cell types or providing optimized thresholds, e.g. log fold change (logFC) from differential analysis.

**Availability and implementation:**

*bootRanges* is freely available in the R/Bioconductor package *nullranges* hosted at https://bioconductor.org/packages/nullranges.

## 1 Introduction

In genomic analysis, to assess whether there is a significant positional association between two sets of genomic ranges, one must choose an appropriate null model (De et al. 2014; Kanduri et al. 2018). Here, we use the term “ranges” to denote a set of genomic features defined by sequence name (e.g. chromosome), starting basepair, ending basepair, and optionally strand and other metadata variables. For example, an enrichment of assay for transposase-accessible chromatin with sequencing (ATAC-seq) peaks near certain genes may indicate a regulatory relationship (Lee et al. 2020), and enrichment of genome-wide association study (GWAS) single nucleotide polymorphisms (SNPs) near tissue-specific ATAC-seq peaks may suggest mechanisms underlying the GWAS trait. Such analyses rely on specifying a null distribution, where one strategy is to uniformly shuffle one set of the genomic ranges in the genome, possibly considering a set of excluded regions where ranges should not be placed (Ogata et al. 2022). However, uniformly distributed null sets will not exhibit the clumping property common with genomic regions. Using an overly simplistic null distribution that doesn’t take into account local dependencies could result in misleading conclusions. More sophisticated methods exist, for example GAT, which allows for controlling confounding factors via segmentation (Heger et al. 2013), and regioneR, which implements a circular shift to preserve the clumping property (Gel et al. 2016). The block bootstrap (Politis et al. 1999) provides an alternative, where one instead generates random sets of ranges by sampling large blocks of ranges from the original set with replacement, as originally proposed for genomic data by Bickel et al. (2010) in Genome Structure Correlation (GSC). Using the block bootstrap is more computationally intensive than simple shuffling, and so GSC implements a strategy of swapping pairs of blocks to compute overlaps, while avoiding a genome-scale bootstrap.

Here, we describe the *bootRanges* software, with efficient vectorized code for performing block bootstrap sampling of genomic ranges stored as GRanges objects in R/Bioconductor (Lawrence et al. 2013). *bootRanges* is part of a modular analysis workflow, where bootstrapped ranges can be analyzed at block or genome scale using tidy analysis with packages including *plyranges* (Lee et al. 2019), and *tidybulk* (Mangiola et al. 2021). We provide recommendations for genome segmentation and block length motivated by analysis of example datasets. We demonstrate how *bootRanges* can be incorporated into complex downstream analyses, including choosing the thresholds during enrichment analysis and single-cell multi-omics. *bootRanges* is distributed as part of the *nullranges* R/Bioconductor package. If directly controlling for nuisance covariates when building background sets is of interest, the sister function *matchRanges* (Davis et al. 2022) may be more appropriate, see the package documentation for an overview of *bootRanges* and *matchRanges*.

## 2 Features

**Figure 1.**
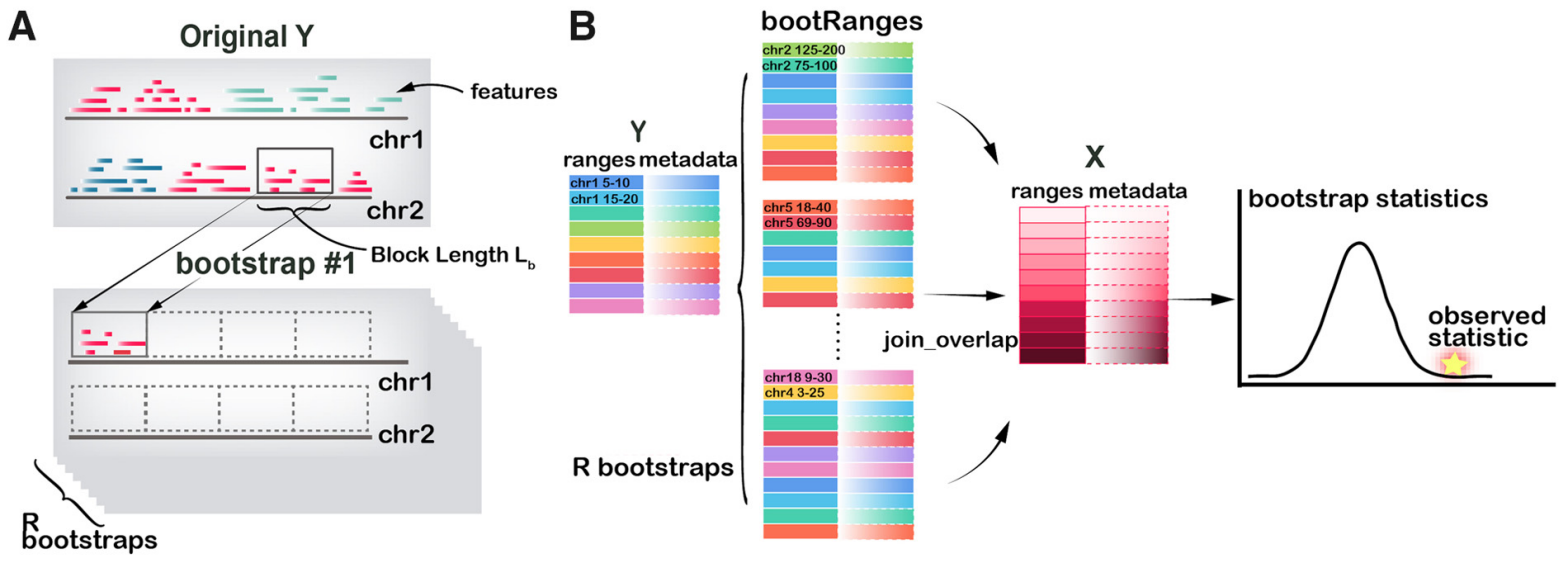
Schematic overview of the segmented block bootstrap for assessing significance of an observed statistic. (A) In each bootstrap sample, new sets of ranges are created by resampling blocks of length $L_{b}$, with replacement, from the original set y. Color represents genome segmentation states, such that blocks may be sampled across chromosomes, from within the same segmentation state. (B) Workflow for testing association between ranges in x and y, e.g. counting the number of overlapping ranges. First, *R* bootstrap samples of y are generated and stored as a *BootRanges* object. Then, the bootstrap distribution of test statistics (e.g. count of overlaps) between x and *BootRanges* is computed. Finally, the observed overlap statistic between x and y is compared to the bootstrap distribution.

*bootRanges* offers a “segmented” block bootstrap: since the distribution of ranges in the genome exhibits multi-scale structure, we follow the logic of Bickel et al. (2010) and consider to perform block bootstrapping within *segments* of the genome, which are more homogeneous in their range density. A simple “unsegmented” block bootstrap is additionally implemented but the segmented version is generally recommended. We consider various genome segmentation procedures, segmenting on gene density, or pre-existing annotations, e.g. Giemsa bands or published segmentations. The genome segments define large (e.g. on the order of ∼1 Mb), relatively homogeneous segments from which to sample blocks (Fig. 1A). Blocks are sampled across segments that are in the same segmentation *state* (see Supplementary Material for details). The input for the workflow is range sets x and y, with optional metadata columns that can be used for computing a more complex test statistic than overlaps. Given a segmentation and block length $L_{b}$, a *BootRanges* object is generated, which concatenates ranges across bootstrap iterations. This *BootRanges* object can be manipulated with *plyranges* to derive the bootstrap distribution of test statistics $\left\{s_{r}\right\}$ and a bootstrap *P*-value: $\frac{1}{R}\sum_{r=1}^{R}I_{\left\{s_{r}>s_{\text{obs}}\right\}}$ (Fig. 1B). The *bootRanges* algorithms are explained schematically in Supplementary Section S1.6.

## 3 Application

### 3.1 Test for association between SNPs and peaks

**Figure 2.**
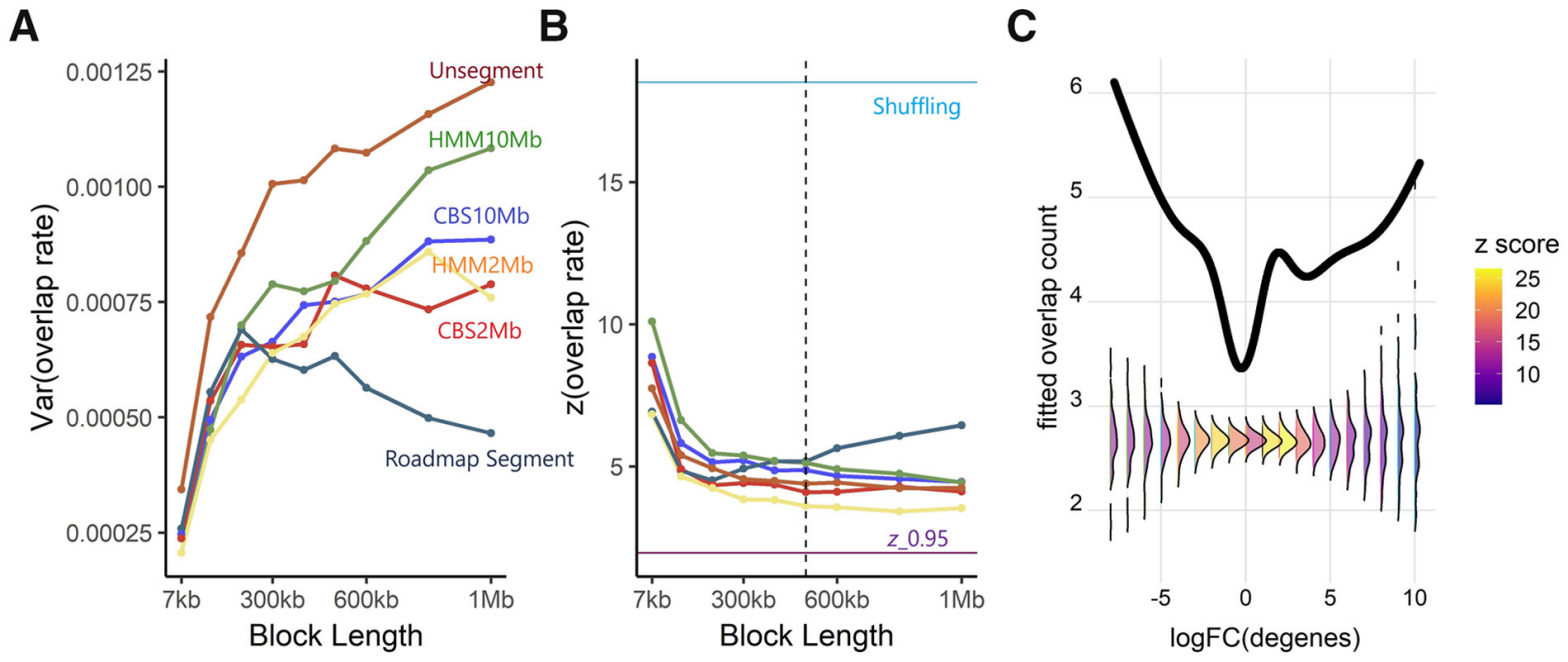
Parameter selection and overlap analysis. (A) Variance of the rate of overlaps and (B) *z* score for the overlap, for different segmentations and $L_{b}$ on the liver dataset. (C) GAM predicted curves for observed (black line) and bootstrapped data (densities), for the overlap count over gene logFC. Conditional densities are colored by the *z* score for the overlap.

We first applied *bootRanges* to determine the significance of the overlap of liver ATAC-seq (Currin et al. 2021) with SNPs associated with total cholesterol, bootstrapping the SNPs to assess significance. While the observed overlap was significant across many combinations of various segmentation methods and $L_{b}$ according to typical *P*-value cutoff, the variance of the bootstrap statistics distribution and the resulting *z* score varied across segmentation choice, though varying more across block length (Fig. 2A and B). The effect of segmentation may be stronger in other contexts. We used the *z* score to measure the distance between the observed statistics and bootstrap distribution in terms of standard deviations. Overlap rate was defined as the proportion of SNPs that had peaks within 10 kb. That the variance of the overlap rate in Fig. 2A for the unsegmented bootstrap was larger than for the segmented cases and increased with $L_{b}$ indicated that the density of ranges varies along the genome and that bootstrapping with respect to a genome segmentation may be a more appropriate choice (Bickel et al. 2010). The decreasing trend using pre-defined segmentation from Roadmap Epigenomics indicated too many short segments, where $L_{b}$ is too close to $L_{s}$ for effective block randomization. To chose an optimal segmentation, we noted those for which the variance of the bootstrap distribution becomes stable as $L_{b}$ increases (Fig. 2A). To choose an optimal $L_{b}$ range, two aspects were considered: (i) we sought a minimum value of a scaled version of the change in the variance of bootstrap statistics distribution across $L_{b}$, as recommended previously (Bickel et al. 2010), and (ii) we assessed whether the distribution of inter-range distances was preserved, when comparing to the original ranges (Supplementary Section S1.5). Here, $L_{s}\approx 2$ Mb and $L_{b}\in [300\text{kb},600\text{kb}]$ was shown to be a good range for segment and block lengths (Supplementary Fig. S2A–C, and Supplementary Section S2.2). The scientific conclusion of this example was that liver ATAC-seq peaks were much closer to total cholesterol SNPs than expected even when placing blocks of SNPs to match a genome segmentation. Shuffling of genomic ranges (Supplementary Section S1.3) resulted in a much higher z=18.5, compared to z≈4 consistent with previous conclusions that shuffling may overestimate significance leading to misinterpretation of enrichment.

### 3.2 Choosing thresholds for enrichment analysis

We demonstrated using *bootRanges* to motivate the choice of data-driven thresholds during enrichment analyses. We tested this on a dataset of differential chromatin accessibility and gene expression (Alasoo et al. 2018; Lee et al. 2020) and the liver ATAC-seq. A generalized linear model with penalized splines was fit to the overlap count over gene logFC, both for the original data and to each of the generated null sets. Conditional densities of splines fit to null sets were computed at various thresholds to reveal how the threshold choice would affect the variance of the bootstrap density and the resulting *z* score (Fig. 2C). The observed enrichment with respect to bootstrapped ranges varies over the logFC threshold. Instead of picking an arbitrary logFC cutoff, these analyses suggested that SNPs with -log10 (*P*-value) > 8 and genes with |logFC|>2 were sets where the *z* score reflected strong separation of the observed statistic from the bootstrap statistic distribution (Supplementary Fig. S3).

### 3.3 Identification of gene-promoter pairs by single-cell multi-omics

We applied *bootRanges* to single cell multiome ATAC-seq and RNA-seq, to assess the correlation (ρ) of log counts for the two modalities for all pairs of genes and peaks, across 14 cell types (pseudo-bulk). Across all genes, we observed $\bar{\rho }=0.33$, which was significantly higher than the bootstrap correlation mean (Supplementary Fig. S4A, ${\bar{\rho }}_{R}=0.007$). As expected, RNA and ATAC measured at local peaks had similar cell-type-specificity. Additionally, the average gene-peaks correlation per gene can be computed and compared to a bootstrap distribution to identify gene-promoter pairs that were significantly correlated across cell types (Supplementary Fig. S4B and C).

### 3.4 Simulation and timing

To assess the accuracy of the bootstrap distribution in capturing the true null distribution, we generated a simulation in which there was no true association between x and y. We compared the bootstrap distribution using *bootRanges* with that using shuffling. Details are provided in Supplementary Section S2.1. Given that there is no true association, we would expect false positive rate (FPR) ≈α, the threshold for significance, if the randomization method was successfully approximating the data generating distribution for y. We found that *bootRanges* could achieve an FPR near α. Shuffling however generated a distribution of statistics with similar mean as the original distribution, but with much lower variance. Therefore, the FPR for shuffling in this simulation was relatively high and would lead to overestimation of the significance of the overlap (Supplementary Fig. S1).

To compare speed, we ran *bootRanges* and GSC on ENCODE kidney and bladder ChIP-seq. The average time to block bootstrap the genome using *bootRanges* was 0.30 s and 0.37 s with overlap computation. A comparable analysis with GSC took 7.56 s.

## 4 Conclusion

*bootRanges* efficiently generates null models of genomic ranges preserving local genomic correlations, and can be used easily in combination with other range-based tools such as *plyranges*. Its versatility allows for the exploration of various hypotheses related to any type of feature in genomic analysis, including investigating gene regulation that is specific to certain cell types.

## Supplementary Material

btad190_Supplementary_Data

## Data Availability

*bootRanges* is distributed in the *nullranges* R/Bioconductor package, while all of the R scripts and data used in this article are available at the following GitHub repository: https://github.com/Wancen/bootRangespaper.
